# Prostatic malakoplakia: a rare case report and literature review

**DOI:** 10.3389/fonc.2026.1896863

**Published:** 2026-09-24

**Authors:** Yuanhao Wang, Gang Mu

**Affiliations:** Department of Urology, The Fourth People’s Hospital of Shenyang, Shenyang, Liaoning, China

**Keywords:** antibiotic therapy, diagnosis, magnetic resonance imaging, Michaelis-Gutmann bodies, prostate cancer, prostatic malakoplakia

## Abstract

Malakoplakia is an extremely rare chronic inflammatory disorder, most commonly affecting the genitourinary tract. Prostatic malakoplakia is particularly uncommon. Its elevated serum prostate-specific antigen (PSA) levels, magnetic resonance imaging (MRI) findings, and clinical symptoms closely mimic those of prostate cancer, leading to a high risk of misdiagnosis. Here we report a 64-year-old male patient presenting with gross hematuria, frequency, and urgency. Laboratory tests revealed elevated inflammatory markers and a serum PSA of 8.72 ng/mL. Multiparametric MRI (mpMRI) showed a Prostate Imaging Reporting and Data System (PI-RADS) score of 4, raising high suspicion of prostate cancer. Transrectal ultrasound-guided prostate biopsy was performed. Histopathology showed diffuse infiltration by histiocytes containing characteristic Michaelis-Gutmann (MG) bodies with typical morphological features on hematoxylin and eosin (H&E) staining. Immunohistochemistry confirmed prostatic malakoplakia and ruled out malignancy. After treatment with piperacillin sodium, the patient’s symptoms resolved completely. Follow-up at one month showed normalization of inflammatory markers and PSA. This article also narrative literature review 90 cases reported in the past 30 years, summarizing the clinical presentation, imaging features, pathological characteristics, diagnosis, differential diagnosis, and treatment strategies for prostatic malakoplakia. In patients with elevated PSA and mpMRI findings suspicious for prostate cancer, if the lesion shows diffuse symmetric distribution, an intact capsule, and delayed periprostatic enhancement, prostatic malakoplakia should be included in the differential diagnosis. Definitive diagnosis depends on pathological identification of MG bodies. Antibiotic therapy appears to be effective in most reported cases, and the prognosis is generally favorable.

## 1 Case presentation

A 64-year-old male was admitted with a 10-day history of gross hematuria. The patient developed painless gross hematuria without obvious triggers, accompanied by urinary frequency and urgency, but no fever or significant dysuria. Past medical history was unremarkable. Admission laboratory tests: Urinalysis showed red blood cells 176/μL (reference range 0–5/μL), white blood cells 156.24/HPF (reference range 0–5/HPF). Complete blood count: white blood cells 13.13×10^9^/L (reference range 3.5–9.5×10^9^/L), neutrophil percentage 81.1% (reference range 40%–75%), absolute neutrophil count 10.65×10^9^/L (reference range 1.8–6.3×10^9^/L). Serum total PSA was 8.72 ng/mL (reference range 0–4 ng/mL). A midstream urine culture was collected on admission, which showed no bacterial growth. Digital rectal examination revealed grade II prostatic enlargement, but no distinct nodules. Contrast-enhanced CT of the urinary tract suggested changes in the left peripheral zone of the prostate and recommended contrast-enhanced MRI (**Figure 1**). Subsequent mpMRI of the prostate showed: a plump prostate measuring approximately 46 mm × 35 mm; poorly defined boundaries among the central, transitional, and peripheral zones with decreased signal on T2WI; high signal intensity in most of the peripheral zone on diffusion-weighted imaging (DWI) with slightly decreased apparent diffusion coefficient (ADC); early post-contrast images showed scattered relatively uniform enhancement in the peripheral zone with a focal area of decreased enhancement on the left, and delayed images showed heterogeneous enhancement of the central and transitional zones; bilateral seminal vesicles were symmetric with no signal abnormalities. Imaging findings suggested possible prostate cancer (PI-RADS 4), but features such as diffuse symmetric distribution in the peripheral zone, intact capsule, and delayed periprostatic enhancement were not entirely typical (**Figure 2**). Given the elevated PSA level and suspicious imaging findings, the patient underwent transrectal ultrasound-guided prostate biopsy. A total of 12 cores were obtained, comprising 10 systematic cores and 2 targeted cores directed at the PI-RADS 4 lesion. All specimens were submitted for histopathological examination. Microscopy showed numerous aggregated histiocytes with abundant eosinophilic cytoplasm, containing round MG bodies measuring 4–10 μm in diameter with a targetoid or “owl’s eye” appearance. Immunohistochemistry demonstrated P63 (+), P504S (−), CD68 (+), S100 (−), and SOX-10 (−), consistent with prostatic malakoplakia (**Figures 3**, **4**). The patient received intravenous piperacillin sodium (2 g twice daily) for 12 days. His clinical symptoms gradually resolved. One month after discharge, serum total PSA decreased to 3.85 ng/mL (reference range 0–4 ng/mL), with normalization of urinalysis and inflammatory markers. No recurrence was observed during the 1 month follow-up.

**Figure 1:**
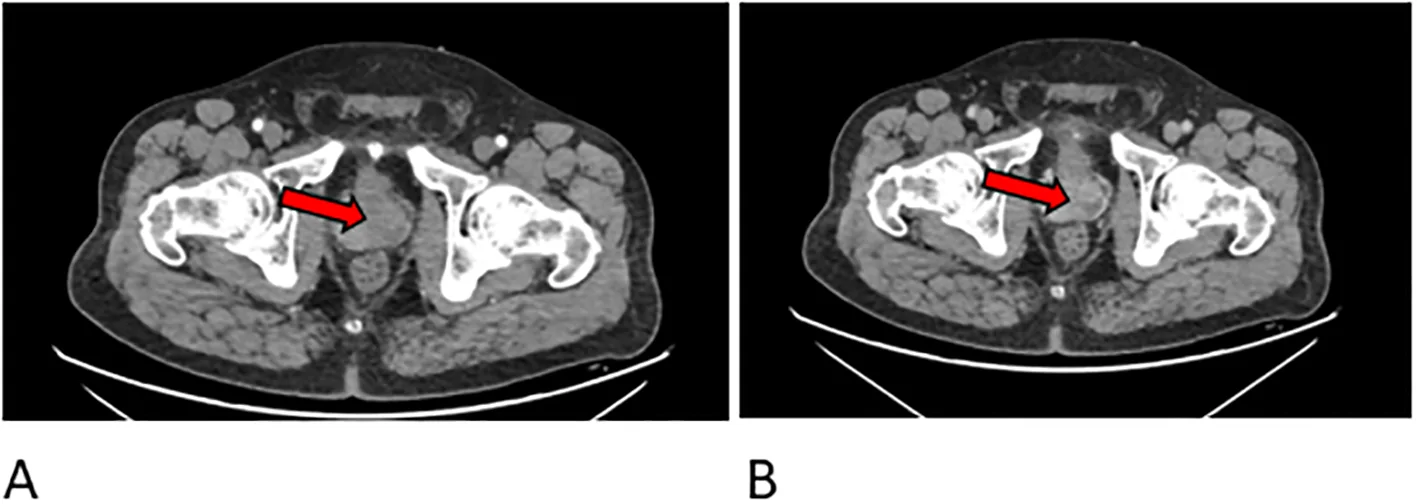
Urinary contrast-enhanced CT [**(A)** arterial phase, **(B)** venous phase]. The red arrow indicates the suspicious area in the left peripheral zone of the prostate.

**Figure 2:**
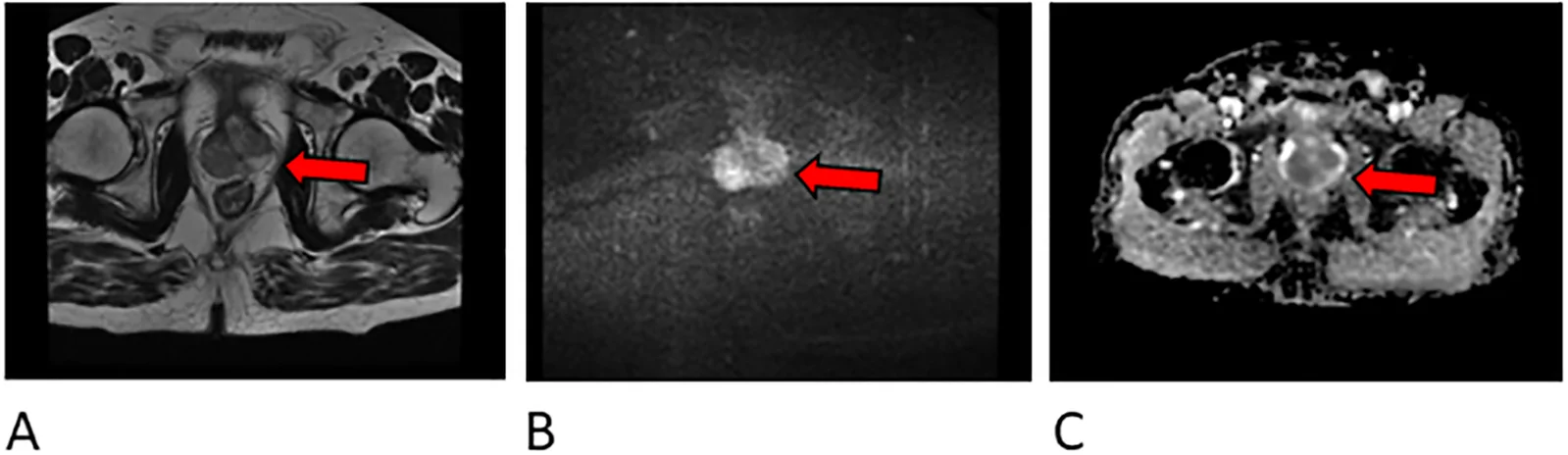
Multiparametric MRI of the prostate. **(A)** Axial T2-weighted imaging (TR/TE = 4500/102 ms): Diffuse indistinct hypointensity involves most of the peripheral zone, with a more prominent focal abnormal signal in the left peripheral zone (red arrow); the zonal boundaries of the prostate are blurred, and the prostatic capsule remains intact without infiltration. **(B)** Axial diffusion-weighted imaging (b = 1000 s/mm²): Corresponding hyperintensity is observed within the abnormal left peripheral zone lesion (red arrow). **(C)** Axial apparent diffusion coefficient (ADC) map: Mildly reduced diffusion signal is noted at the lesion site (red arrow). The overall mpMRI findings were categorized as PI-RADS 4. Despite the suspicious malignant-grade score, diffuse symmetric peripheral zone involvement and an intact prostatic capsule suggested an inflammatory lesion (prostatic malakoplakia) rather than primary prostate cancer.

**Figure 3:**
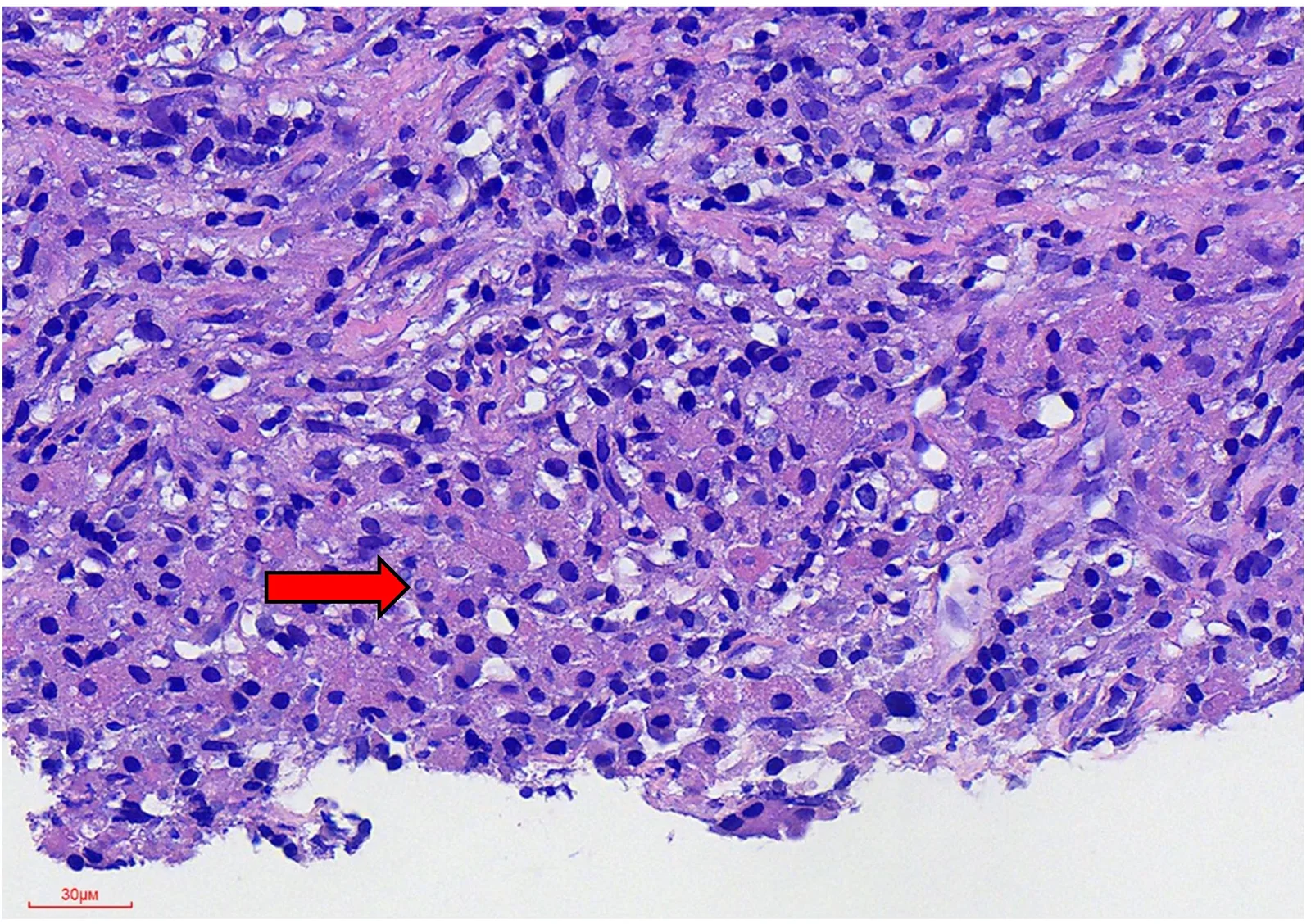
Histopathological findings (HE staining, ×400). Numerous histiocytes with abundant eosinophilic cytoplasm are seen, containing round, targetoid, “owl’s eye”-like Michaelis-Gutmann bodies (red arrows).

**Figure 4:**
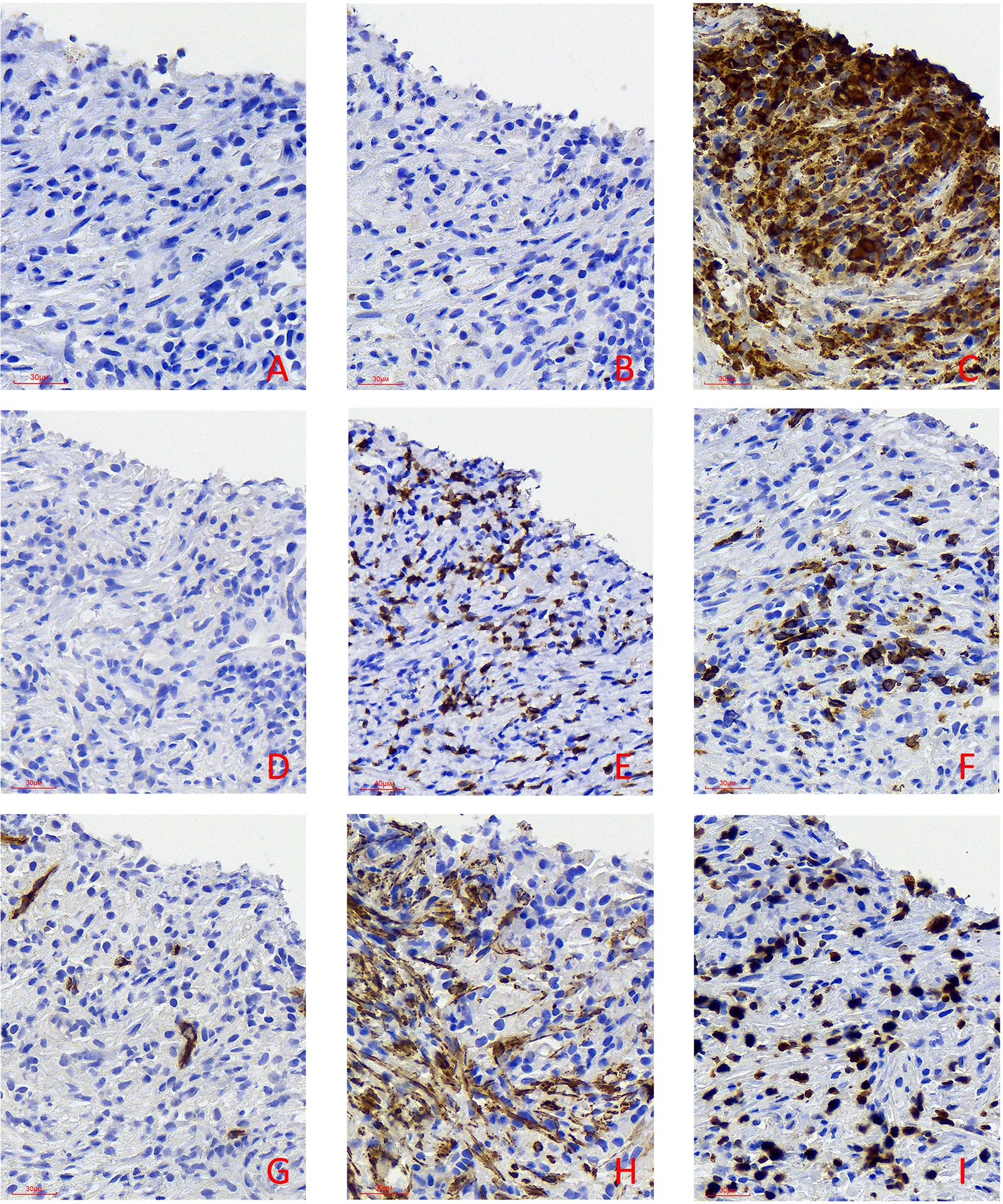
Immunohistochemical staining of the prostatic lesion (×400). **(A)** High molecular weight cytokeratin (CKH): positive in epithelial components; **(B)** P63: positive in basal cells, supporting an intact basal cell layer; **(C)** CD68: strongly positive in lesional histiocytes, confirming histiocytic lineage; **(D)** pan-CK: positive in epithelial cells; **(E)** CD3: positive in infiltrating T lymphocytes; **(F)** CD20: positive in infiltrating B lymphocytes; **(G)** CD34: positive in vascular endothelial cells; **(H)** Smooth muscle actin (SMA): positive in stromal smooth muscle cells; **(I)** Ki67: positive in approximately 30% of inflammatory infiltrating cells (predominantly lymphocytes and reactive histiocytes), rather than epithelial cells.

## 2 Discussion

Prostatic malakoplakia is an extremely rare chronic inflammatory disorder of the genitourinary tract, first defined by Carruthers in 1959 (1), with fewer than 100 cases reported in the English literature to date (2, 3). The disease predominantly affects elderly males with a median age around 67 years (2, 4). Previous literature has reported that serum PSA levels in patients with prostatic malakoplakia are frequently elevated to varying degrees. The range of median PSA across studies was 7.5–16.05 ng/mL (4–6), with the majority of patients having PSA >4 ng/mL. The mechanism of PSA elevation may be related to inflammatory disruption of the prostatic epithelial barrier, leading to PSA leakage into the circulation. Regarding etiology, the disease has been closely associated with Escherichia coli (E. coli) infection, which has been identified in over two-thirds of reported cases. This association has been documented in both prostatic malakoplakia (4, 5) and renal malakoplakia (7). However, not all patients yield positive culture results. In the present case, midstream urine culture was negative, a finding that does not contradict the literature-based E. coli association. Acosta et al. (4) reported positive microbiological findings in only approximately 69% of 49 patients (of which 64% were E. coli). Moreover, when the infection focus is located within the prostatic parenchyma rather than the urinary lumen, the sensitivity of urine specimens is reduced. Therefore, although our case did not provide direct microbiological evidence for E. coli infection, the literature data consistently support this association. In clinical practice, antibiotic selection should be individualized based on the patient’s clinical response and, when available, bacteriological culture and susceptibility results. Diabetes mellitus, organ transplantation, and long-term immunosuppression are recognized predisposing factors (3, 8). Clinical manifestations are nonspecific, overlapping with prostatitis and prostate cancer. Common symptoms include LUTS (urgency, frequency, weak stream, acute urinary retention), gross hematuria, recurrent UTIs, and occasionally fever and sepsis (2, 4, 6). Some patients are asymptomatic and detected only through PSA screening (5, 9). A summary of the clinical features of the 90 reviewed cases is provided in **Table 1**. Our patient presented with gross hematuria and irritative voiding symptoms, without severe obstruction or systemic symptoms, consistent with the literature.

**Table 1:** Summary of Prostatic Malakoplakia Cases Reported in the Past 20 Years.

Reference	Year	Age	Symptoms	PSA(ng/ml)	Imaging features	MG bodies / staining	Urine culture	Treatment	Remarks
Ren Y, et al. (10)	2024	58–74 (median 71)	Mostly asymptomatic	Median 10.40 (1.74–63.42)	MRI: T1 high / T2 low, PI-RADS 4–5	PAS+, calcium stain+	1 case E. coli	Observation / TURP	1 case with abnormal pelvic lymph node morphology
Repassy DL, et al. (11)	2002	56	Urinary retention	Mildly elevated → 0.3 post-op	Ultrasound: heterogeneous hypoechoic	+	E. coli	Radical prostatectomy	Concurrent prostate adenocarcinoma
Bidot S, et al. (12)	2020	61	Obstructive LUTS	1.83	MRI: diffuse edema, restricted diffusion	Visible on H&E	E. coli	Ceftriaxone + TURP	Post−liver transplant, diabetes, immunosuppressed
Medlicott S, et al. (13)	2016	58–68	Obstruction / infection / retention	1.6–30.29	Enlarged prostate or nodule	PAS+, von Kossa+	2 cases E. coli	Radical prostatectomy / conservative	All cases with concurrent prostate adenocarcinoma (Gleason 6–7)
Guner G, et al. (14)	2012	72	LUTS, acute retention	19.23→100→30.19	CT: enlarged with hypodense areas	PAS+, von Kossa+	Acute prostatitis (unspecified)	Radical prostatectomy	Concurrent prostate adenocarcinoma (Gleason 6)
Geng S, et al. (15)	2025	68	Dysuria, nocturia	1.81	PI-RADS 5, T2 low / DWI high / ADC low	PAS+	Urine WBC↑, culture negative	Levofloxacin + thulium laser enucleation	Post−heart transplant, diabetes, immunosuppressed
Kahraman D, et al.(case 1) (4)	2014	70	Burning, discomfort	14.1	Enlarged, palpable nodule	PAS+	E. coli	Antibiotics + open resection	–
Kahraman D, et al. (case 2) (4)	2014	69	Burning, discomfort	100	Not specified	PAS+	E. coli	Antibiotics	–
Rances OT, et al. (16)	2023	69	Asymptomatic	6.11	PI-RADS 3	+	Post−biopsy UTI	Robotic radical prostatectomy	Concurrent prostate adenocarcinoma (Gleason 3+4)
Yang Q, et al. (17)	2024	NR	Dysuria, fever	NR	Peripheral zone nodule, suspicious for cancer	PAS+, H&E+	Blood: E. coli	Meropenem → cefuroxime	Secondary sepsis, multiorgan dysfunction
Kitzing YX, et al. (18)	2016	49	Recurrent prostatitis, PSA↑	Elevated (NS)	MRI: T2 low, restricted diffusion, marked enhancement	+	Unknown	Unknown	Individual case mentioned in review
Heah NH, et al. (19)	2017	75	Asymptomatic	16.6→21.76	PI-RADS 5 (right apex) & 4 (left mid-gland)	H&E "owl's eye" MG bodies	E. coli UTI + bacteremia	Antibiotics	Secondary bacteremia
Velasquez MC, et al. (20)	2018	63	LUTS	10→18.26	PI-RADS 4, seminal vesicle invasion	von Kossa+, Prussian blue+, PAS+	E. coli UTI	Antibiotics	Post−renal transplant, immunosuppressed
Gorgel SN, et al. (2)	2011	65	Acute retention	Preop 6.5 → postop 1.5	DRE hard nodule; ultrasound bilateral hypoechoic	PAS+	E. coli	TURP + ceftazidime	–
Dale RT, et al. (21)	2015	66	Retention (post−biopsy)	8.0	Left mid-gland 4.5×2 cm, right base 2.1×1.5 cm, seminal vesicle invasion	Prussian blue+, von Kossa+, PAS/D+	E. coli UTI	Robotic radical prostatectomy	Post−biopsy UTI, retention
Wagner D, et al. (22)	2007	66	Asymptomatic (abnormal DRE)	5.33 (17.7 during pyelonephritis)	TRUS: enlarged with hypoechoic areas	von Kossa+, CD68+ (PAS not done)	History of acute right pyelonephritis (E. coli)	Antibiotics	Type 2 diabetes
Subbaraya S, et al. (case 1) (23)	2021	75	Acute retention, LUTS	4.6	mpMRI: peripheral zone thickening, diffuse T2 low, ADC low	PAS+, von Kossa+	E. coli	Ciprofloxacin + TURP	–
Subbaraya S, et al. (case 2) (23)	2021	63	Acute retention, LUTS	11.3	TRUS: 57 cc; DRE: grade II hard, left nodule	+	E. coli	Ciprofloxacin + tamsulosin	Type 2 diabetes
Rezaee ME, et al. (24)	2020	57	LUTS (IPSS 20)	3.1-4.7	PI-RADS 5, extensive EPE, neurovascular bundle invasion	MG bodies (–), eosinophilic histiocytes	Urinalysis negative	Robotic radical (non−nerve−sparing)	–
Zeng HJ, et al. (3)	2025	46–83 (median 62)	7/11 LUTS; 4/11 asymptomatic	Median 16.05 (IQR 7.77–31.57)	PI-RADS 4/5; diffuse symmetric (5/11); early enhancement + delayed periprostatic enhancement (11/11)	+	Often E. coli–associated	TMP−SMX/fluoroquinolones effective	2 diabetes, 2 hypertension, 1 with low−volume Gleason 3+4 prostate cancer (<5%)
Zhao H, et al. (25)	2018	43	Fever	148→48→normal	Ultrasound: enlarged with mass; MRI suggested tumor	PAS+, CD20+, CD68+	Blood/urine culture negative	Meropenem (>1 month)	Hypertension
Arena F, et al. (26)	2001	58	Urethral discharge, retention, fever	Unknown	CT: enlarged (5×6.5×7 cm), abscess, left seminal vesicle invasion	PAS+, Prussian blue+, von Kossa+, H&E+	E. coli (>10^5 CFU)	TURP drainage + TMP−SMX 8 weeks	Concurrent diabetes; secondary prostatic abscess
Guo S, et al. (5)	2024	80	Frequency, urgency, dysuria (10 years), fever, retention	tPSA 15.62, fPSA 1.32, ratio 0.08	PI-RADS 5, peripheral + transition zones	PAS+, Prussian blue+, CD68+	Unknown	Antibiotics (1 month)	–
Spiker M, et al. (1)	2025	67	Painless hematuria, weight loss, fever	Undetectable on admission (prior 13)	Paraprostatic cystic mass (6.4×5→8.8×7.7 cm), bilateral hydronephrosis	Visible on H&E, von Kossa+	E. coli (urine + blood)	Levofloxacin (3 months)	Concurrent prostate adenocarcinoma (Gleason 4+4); secondary sepsis
Acosta AM, et al. (6)	2022	38–77 (median 67)	71% LUTS	Median 7.5 (0.7–49.1), 94% ≥4	12% abnormal DRE / MRI PI-RADS 4–5	von Kossa+ 92%, PAS+ 96%	69% culture positive (64% E. coli)	Antibiotics or surgery	49% had concurrent malignancy (37% prostate cancer); 26% diabetes/insulin resistance; 6% solid organ transplant; 1 HIV

Malakoplakia was first reported by Michaelis and Gutmann in 1902 from a bladder biopsy specimen (27). The disease primarily involves the genitourinary tract, with pathological hallmarks including large accumulations of histiocytes (von Hansemann cells) containing characteristic MG bodies within the cytoplasm (5). On mpMRI, prostatic malakoplakia frequently presents as low signal on T2WI, high signal on DWI, low signal on ADC, with PI-RADS scores typically 4–5, closely resembling prostatic adenocarcinoma (5). This diagnostic challenge is not unique to the prostate; similar imaging pitfalls have been reported in rectal malakoplakia (9). Such findings often lead to misdiagnosis and unnecessary invasive procedures, as has been observed in both prostatic (4) and bladder malakoplakia (10).

Distinguishing prostatic malakoplakia from prostate cancer on imaging is clinically challenging. Zeng et al. (5) summarized mpMRI features of 11 pathologically confirmed cases and pointed out key differentiating points: lesions predominantly located in the peripheral zone, with approximately 45.5% showing diffuse symmetric distribution. The prostatic capsule remains intact, with a bulging rather than infiltrative appearance. Slightly high signal on T1WI may be related to calcium and iron deposition within MG bodies. Dynamic contrast-enhanced imaging showed early enhancement in all cases, with 72.7% demonstrating persistent enhancement, and all showed delayed periprostatic enhancement, suggesting an inflammatory reaction in surrounding tissues. In addition, these patients had significantly larger peripheral zone volumes than those with prostate cancer. Therefore, for PI-RADS 4–5 lesions, if the above features are present, malakoplakia should be highly suspected, thereby avoiding unnecessary radical surgery (5, 9, 10).

Definitive diagnosis depends on pathological identification of MG bodies, the characteristic markers of malakoplakia. MG bodies are round intracytoplasmic inclusions in histiocytes, measuring 4–10 μm in diameter, with a targetoid or laminated “owl’s eye” appearance (5, 27) containing incompletely degraded bacterial fragments, mucopolysaccharides, calcium, and iron. They stain positively with PAS, von Kossa, and Prussian blue stains (2, 4). In our patient, PAS, von Kossa, and Prussian blue staining were not performed due to institutional limitations. Nevertheless, the diagnosis is strongly supported by the typical morphology of MG bodies on H&E staining (targetoid/owl’s eye appearance), combined with CD68-positive immunohistochemical findings. Although PAS, von Kossa, and Prussian blue staining are reported as standard special stains for MG bodies in the literature (2, 4), the cornerstone of MG body diagnosis remains morphological recognition on H&E staining (2, 5); special stains serve as ancillary confirmatory tools and are not mandatory for diagnosis.

Immunohistochemistry demonstrated P63 positivity with P504S negativity, indicating an intact basal cell layer and effectively excluding invasive prostatic adenocarcinoma (3, 9); strong CD68 positivity in lesional histiocytes, consistent with histiocytic-macrophage lineage (4, 5); and negativity for S100 and SOX-10, excluding melanocytic tumors and neuroendocrine neoplasms (6). Ki67-positive cells were predominantly localized to inflammatory infiltrates and reactive histiocytes (approximately 30%), rather than epithelial components, arguing against malignant proliferation. This combined immunohistochemical profile, together with the typical H&E morphology, provides robust evidence for the diagnosis of prostatic malakoplakia. It should be emphasized that coexisting prostatic adenocarcinoma has been documented in cases of malakoplakia in the literature (4, 11–14). Therefore, even when imaging and biopsy pathology support benign malakoplakia, the possibility of concurrent carcinoma should not be overlooked. In our patient, a 12-core biopsy (10 systematic cores + 2 targeted cores directed at the PI-RADS 4 lesion) was performed, and all specimens showed no evidence of malignancy; the P504S-negative and P63-positive pattern further supported a benign diagnosis. Nevertheless, given the sampling limitations of a single biopsy, we recommend regular PSA monitoring (e.g., every 3–6 months) for such patients. If PSA rises again or new suspicious imaging lesions appear, repeat biopsy should be actively considered to exclude the possibility of prostate cancer coexisting with malakoplakia. Treatment is individualized based on lesion extent, symptoms, and comorbidities. First-line therapy uses antibiotics that accumulate in macrophages, such as β-lactams, trimethoprim-sulfamethoxazole, fluoroquinolones, and cephalosporins (4, 7, 8). Although initial experience with macrophage-penetrating antibiotics derived partly from renal malakoplakia (7), subsequent studies in prostatic malakoplakia have confirmed similar efficacy (4). Most patients achieve symptom relief, normalization of PSA and inflammatory markers after a prolonged course of antibiotics (3, 5). Surgical treatment (transurethral resection of the prostate, enucleation) is reserved for cases with severe obstruction, abscess formation, or failure of medical therapy (2, 6, 11). It should be noted that the antibiotic course in this case was relatively short (12 days) compared with the prolonged courses typically recommended for malakoplakia (3–5). Nevertheless, the patient showed significant clinical improvement and a marked decrease in PSA from 8.72 ng/mL to 3.85 ng/mL at 1 month. However, the follow-up period was limited to 1 month, and the patient did not return for further evaluation thereafter. Therefore, the durability of the treatment response and long-term outcomes remain unknown, which constitutes a limitation of this report. Our review of 90 cases suggests a favorable clinical remission rate with targeted antibiotic therapy. Prostatic malakoplakia is a benign process with an indolent course and a generally favorable long-term prognosis; no well-documented malignant transformation has been reported (4, 15).

## 3 Conclusion

Prostatic malakoplakia is a rare benign inflammatory condition that closely mimics prostate cancer clinically and radiologically, leading to a high misdiagnosis rate. Key diagnostic clues include elevated PSA, mpMRI showing PI-RADS 4–5, accompanied by diffuse symmetric distribution in the peripheral zone, intact capsule, and delayed periprostatic enhancement. Definitive diagnosis rests on pathological identification of MG bodies combined with immunohistochemistry. Literature reports indicate that prostatic malakoplakia is closely associated with E. coli infection, and antibiotic therapy targeting this pathogen has shown favorable clinical responses in multicenter studies (3–5). However, urine culture was negative in the present case; therefore, antibiotic selection should be individualized. Increased awareness of this entity may improve diagnostic accuracy, help reduce unnecessary invasive procedures, and optimize patient management.

## Data Availability

The original contributions presented in the study are included in the article/supplementary material. Further inquiries can be directed to the corresponding author.
